# ASO Author Reflections: Decreased Survival from Secondary Breast Cancer in Women of All Ages

**DOI:** 10.1245/s10434-021-10399-y

**Published:** 2021-07-19

**Authors:** Candice A. M. Sauder, Theresa H. M. Keegan

**Affiliations:** 1 Division of Surgical Oncology, Department of Surgery, University of California, Davis Medical Center, 4501 X Street, Suite 3010, Sacramento, CA 95817 USA; 2 Comprehensive Cancer Center, University of California, Davis Medical Center, Sacramento, CA USA; 3 Center for Oncology Hematology Outcomes Research and Training (COHORT), Division of Hematology and Oncology, University of California Davis School of Medicine, Sacramento, CA USA; 4 Division of Hematology and Oncology, Department of Internal Medicine, Davis Medical Center, University of California, Sacramento, CA USA

## Past

Secondary malignancies account for 16% of all new cancer diagnoses in the USA, with breast cancer (BC) being the most common in women over 15 years of age.^1^,^2^ In adolescent and young adults (AYA, 15–39 years), secondary BCs have been shown to have distinct characteristics and worse survival than primary BC.^3^,^4^ However, the characteristics and survival of middle- (40–64 years) and older-aged (≥ 65 years) women with secondary BC were unknown.

## Present

We used the California Cancer Registry to retrospectively examine secondary (versus primary) BC for AYA, middle-aged, and older women. Our data showed decreased survival for secondary BC at all ages, but this difference diminished with age [15–39 hazard ratio (HR) 2.09, 95% confidence interval (CI) 1.83–2.39; 40–64 HR 1.51; CI 1.44–1.58, ≥ 65 HR 1.14; CI 1.10–1.19].^5^ Additionally, our data showed that, for middle-aged Asian/Pacific Islander and Hispanic women, who traditionally have superior survival after primary BC, the impact of secondary BC on decreased survival was substantial. Furthermore, secondary BC survival differences by tumor receptor status were evident in middle- and older-aged women, with the largest impact seen in hormone receptor-positive, human epidermal growth factor receptor 2 (HER2)-negative disease. As a result, more individualized treatment needs to be considered in women with secondary BC, especially those with hormone receptor-positive BC who often are considered to have better prognosis after primary BC.

## Future

Our analysis builds on characteristics and outcomes of secondary BC observed previously in the AYA population^3^,^4^ to include middle- and older-aged women, who accounted for the vast majority of our cohort and BC survivors overall. Treating secondary BC is likely complicated by the unique genetic make-up of the tumors and prior treatment regimens received. These factors should be evaluated in more depth to determine whether the differences seen in survival in the secondary BC population should lead to augmentation of treatment algorithms in this specific population.
